# *Rehmannia glutinosa RgMATE35* Participates in the Root Secretion of Phenolic Acids and Modulates the Development of Plant Replant Disease

**DOI:** 10.3390/plants13213007

**Published:** 2024-10-28

**Authors:** Yanhui Yang, Bingyang Guo, Yan Jin, Mingjie Li, Zichao Wang, Jiaqi Zhao, Haiqin Ma, Tongyu Wu, Zhongyi Zhang

**Affiliations:** 1 School of Bioengineering, Henan University of Technology, Lianhua Street 100, Zhengzhou High-Technology Zone, Zhengzhou 450001, China; 2022920145@stu.haut.edu.cn (B.G.); jinyan@stu.haut.edu.cn (Y.J.); zcwang@haut.edu.cn (Z.W.); zhaojiaqi@stu.haut.edu.cn (J.Z.); 221170400310@stu.haut.edu.cn (H.M.); 221170400128@stu.haut.edu.cn (T.W.); 2 College of Crop Sciences, Fujian Agriculture and Forestry University, Jinshan Road, Cangshan District, Fuzhou 350002, China

**Keywords:** phenolic secretion, multidrug and toxic compound extrusion protein, replant disease, transport activity, autotoxic injury

## Abstract

Phenolic allelochemicals from root exudates dominate rhizosphere formation, lead to autotoxicity in plants subjected to continuous monoculture (CM) stress and induce the emergence of replant disease. However, the regulatory mechanisms governing the transport of phenolics from plant roots to the rhizosphere remain poorly understood. A potential phenolic efflux transporter from *Rehmannia glutinosa*, designated RgMATE35, has been preliminarily characterized. The objective of this study was to elucidate the molecular function of RgMATE35 in the secretion of phenolics and to investigate its role in the development of plant replant disease using quantitative real-time PCR (qRT-PCR), genetic transformation, HPLC-Q-TOF-MS and other analytical techniques. A tissue expression pattern analysis of *RgMATE35* revealed that it is highly expressed in plant roots. Transient expression analysis confirmed the localization of the protein in plasma membranes. An assessment of the transport activity of RgMATE35 in *Xenopus oocytes* indicated that it plays a role in facilitating the efflux of labeled ferulic acid ([^2^H_3_]-FA) and trans-*p*-coumaric acid [^2^H_6_]-*p*CA. The results of functional studies in *R. glutinosa* demonstrated that RgMATE35 positively mediates the secretion of FA and *p*CA from plant roots into the rhizosphere. A molecular and physiological analysis of *RgMATE35* transgenic plants subjected to CM stress revealed that the overexpression or repression of *RgMATE35* resulted in notable changes in the degree of autotoxic injury in plants. These findings demonstrate that RgMATE35 plays a positive role in the development of replant disease through the secretion of phenolic acids from plant roots. They also provide a fundamental framework for elucidating the molecular regulatory mechanism through which MATEs regulate replant disease through the root secretion of allelochemicals.

## 1. Introduction

Phenolics represent one of the most extensively researched groups of allelopathic compounds [1,2,3,4]. These compounds play a crucial role in regulating plant growth and development through their complex interactions with soils and microbial communities [5,6,7]. When plants are subjected to continuous monoculture (CM) stress, the phenolics released from plant root exudates into soils determine the formation of the rhizosphere and lead to allelopathic autotoxicity, which has been identified as a factor in the emergence of plant replant disease [2,4,8,9]. The disease presents a significant challenge to the cultivation of numerous plants, impeding the advancement of agricultural sustainability [9,10,11,12]. To date, many allelopathic phenolics, particularly phenolic acids, have been identified in root exudates from a diverse range of plants, including *Rehmannia glutinosa* [13], *Radix pseudostellariae* [14], *Oryza sativa* [15], *Cucumis sativus* [16], and *Panax quinquefolium* [17]. Previous research has primarily focused on the identification of specific phenolics present in plant root exudates, as well as the evaluation of their physiological activities and the assessment of autotoxic ecological effects [14,18]. Earlier studies have demonstrated that phenolic allelochemicals released from roots play a crucial role in autotoxicity, which is a key factor in the development of replant disease [2,4,14]. However, the precise mechanisms through which phenolics are released from plant roots into the rhizosphere remain to be elucidated. It is of great importance to explore the molecular mechanism of the allelopathic phenolic transport from plant roots to the rhizosphere in order to gain a full understanding of the disease development and address the challenges of the disease.

Multidrug and toxic compound extrusion (MATE) proteins constitute an important family of membrane-localized transporters that actively facilitate the transport of diverse substrates (including secondary metabolites) in all living organisms [19,20,21,22]. In plants, these proteins in plants are mostly composed of 9–12 transmembrane (TM) helical structures and facilitate the transport of substrates across the membrane via the exchange of cations such as H^+^/Na^+^ ions, thereby performing a range of biological functions [19,20,23]. It has been demonstrated that some MATEs are involved in the efflux of phenolics, citrates, and triterpenes, and play a role in many plant biological processes, including disease development and resistance, response to various stress, autotoxin detoxification, and nutrient homeostasis [18,19,21,22,23,24]. The *Arabidopsis thaliana* MATE transport AtDTX18 was elucidated to function as an efflux carrier of endogenous hydroxycinnamic acid amides in plants, with a positive regulatory effect on pathogen defense [24]. The *A. thaliana* MATE transport AtEDS5 was identified as a salicylic acid efflux transporter, which plays a role in SA secretion and the mediation of the pathogen response [25,26]. Two *O. sativa* MATEs, OsPEZ1 and OsPEZ1, were identified as functioning as phenolic acid efflux transporters and as mediators of effective iron uptake [27,28]. The *Sorghum bicolor* (SbMATE) and *Glycine Max* GmMATE13 were both identified as participating in citrate efflux and contributing to aluminum resistance [20,29]. The *Cucumis melo* CmMATE1 and *Citrullus lanatus* ClMATE1 have been demonstrated to facilitate the transport of triterpenes from the roots into the soil, thereby enhancing plant adaptation [30]. Given the pivotal role of phenolics in the development of replant disease, a detailed understanding of the molecular mechanisms underlying phenolic efflux transporters is essential for the effective regulation and alleviation of the disease. It is therefore crucial to investigate the relationship between the transport function of MATEs for allelopathic phenolics and the development of replant disease.

*R. glutinosa*, a perennial herbaceous plant, belongs to the family Orobanchaceae, and is extensively cultivated for its tuberous roots, which possess many pharmacologically active compounds [9,13]. The crop, when subjected to CM stress, results in the development of severe replant disease, which is characterized by the release of phenolic allelochemicals into the soil. This phenomenon manifests as a typical allelopathic injury syndrome, comparable to that observed in other crop species [2,8,16,17]. It has been demonstrated that the primary allelopathic autotoxins derived from the root exudates of *R. glutinosa* are phenolics, including ferulic acid (FA) and trans-*p*-coumaric acid (*p*CA) [2,13]. A previous study identified a potential phenolic efflux MATE transporter from *R. glutinosa*, designated RgMATE35. The transporter was predicted to possess 12 transmembrane domains and localize in the plasma membrane [31]. Moreover, this transporter exhibited a positive correlation with the gene expression and phenolic release in *R. glutinosa* hairy roots [31]. It seems reasonable to suggest that RgMATE35 may be involved in the release of allelopathic phenolics and the modulation of the development of plant replant disease. Accordingly, this study employed *R. glutinosa* as a model plant to investigate the molecular mechanism of the MATE-mediated phenolic release and replant disease formation. To test this hypothesis, a detailed investigation was initiated into the molecular function of RgMATE35 in the context of allelopathic phenolic acid efflux, both in vitro and in vivo. Subsequently, an investigation was conducted to ascertain the biological role of RgMATE35 in *R. glutinosa* subjected to CM stress. This contributes to a better understanding of the regulatory mechanisms underlying the secretion of allelopathic phenolics in the development of plant replant disease.

## 2. Results

### 2.1. Expression Pattern Analysis of RgMATE35 in R. glutinosa

To assess the quality of the isolated RNA samples, the RNA integrity number (RIN) values, and the ratios (18S/28S) of 28S ribosomal RNA (rRNA) and 18S rRNA were determined for the different tissues of *R. glutinosa.* As shown in Table S1 of Supplementary File S1 and Figure S1 of Supplementary File S2, the RIN values were not lower than 7.5 in all samples, and their ratios (28S/18S) of 28S ribosomal RNA (rRNA) and 18S rRNA were greater than 2.0. These results corroborated that the RNA samples were of high integrity, as previously reported [32]. Moreover, the purity of the RNA samples was evaluated. The measured optical density (OD) ratios (OD_260_/OD_280_) between 260 and 280 nm from these RNA samples were approximately 2.0, indicating minimal contamination by proteins, phenol and other impurities, as previously reported [33]. Furthermore, the OD ratios (OD_260_/OD_230_) between 260 and 230 nm were found to be greater than 2.0, indicating high purity in the RNA samples, as previously reported [33]. Therefore, these results indicated that the RNA samples were of optimal quality and met the criteria for the subsequent experiments.

**Figure 1:**
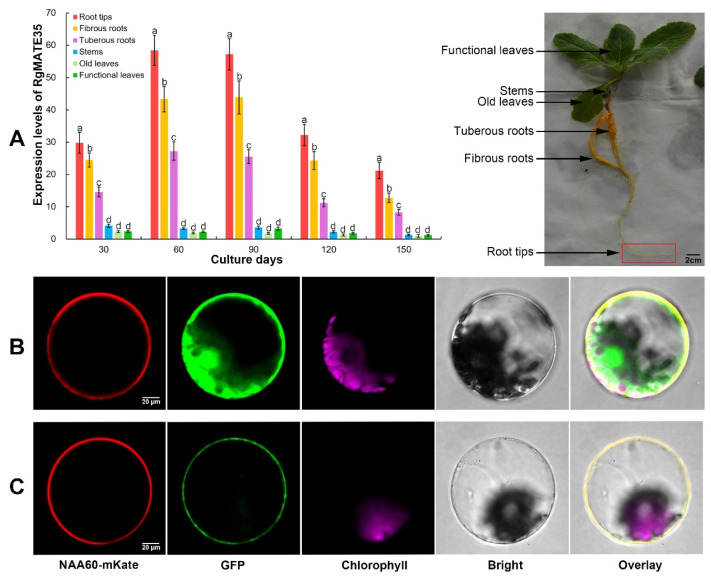
(**A**) The *RgMATE35* tissue expression profiles of *R. glutinosa* during these cultivation periods and a photograph depicting plant tissues at the 30-day cultivation mark. The error bars represent the standard error, and different lowercase letters indicate significance at the 0.05 level among the tissues (*n* = 3). The figure also includes (**B**) GFP (control) and (**C**) RgMATE35-GFP protein subcellular localization in the species protoplasts (note: the first column shows the plasma membrane marker NAA60-mKate fluorescence patterns; the second column depicts GFP fluorescence patterns; the third column displays chloroplast autofluorescence; the fourth column presents a bright-field image of each protoplast; and the fifth column shows an overlay of the fluorescent images). These images are representative of four independent experiments.

To assess the expression profile of *RgMATE35*, its transcript abundance in various tissues of *R. glutinosa* across distinct cultivation stages was quantified through quantitative real-time PCR (qRT-PCR) (Figure 1A and Table S2 of Supplementary File S1). In general, the relative tissue expression levels of *RgMATE35* at these stages demonstrated comparable patterns of significant difference. Specifically, the expression level of *RgMATE35* in roots was found to be more than 3-fold that observed in stems and leaves. For instance, the difference in tissue levels between 60 and 90 days was more pronounced than that observed in other cultivation periods. For example, the levels in roots at 60 days of cultivation are approximately 14-fold those in stems and leaves. Furthermore, *RgMATE35* expression in root tips exhibited the highest levels among the various root parts during the cultivation periods. For instance, the expression level in root tips was at least 2-fold that in tuberous roots at all of these stages. The results indicated that *RgMATE35* exhibited significant expression efficiency predominantly in the roots of *R. glutinosa*, specifically concentrated in the root tips.

### 2.2. Subcellular Localization of RgMATE35

To determine the subcellular localization of RgMATE35, the CaMV35S:RgMATE35-GFP construct (Figure S2A of Supplementary File S2) or the CaMV35S:GFP empty vector (control) was transiently co-transformed in *R. glutinosa* protoplasts with CaMV35S:NAA60-mKate (plasma membrane marker). Green fluorescence from the CaMV35S:GFP construct was observed throughout the plasma membrane, cytoplasm, nucleus, and other cell organelles (Figure 1B). The expression of NAA60-mKate resulted in the display of red fluorescence at the plasma membrane (Figure 1B). Moreover, the distribution of the GFP signal was found to be analogous following the expression of the RgMATE35-GFP construct. Furthermore, the merged signals of NAA60-mKate and RgMATE35-GFP displayed near-identical patterns (Figure 1C). These results indicated that RgMATE35 is localized in plasma membranes, thereby confirming its classification as a plasma membrane protein.

### 2.3. Assessment of RgMATE35 Efflux Activity in Xenopus oocytes

**Figure 2:**
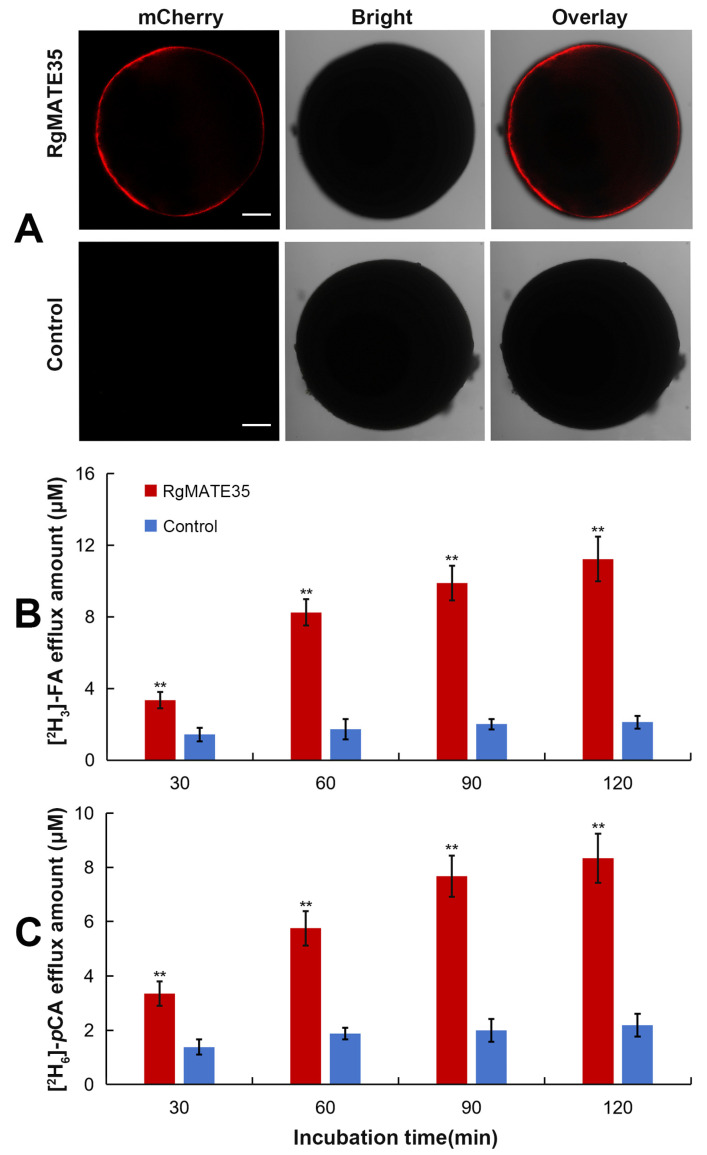
Expression and transport activity analysis of RgMATE35 in *X. oocytes*. (**A**) The expression of RgMATE35-mCherry protein and mCherry (control) is shown in plasma membranes (scale bar = 200 μm); (**B**,**C**) efflux amounts of phenolic acids were tested by RgMATE35 transport during these incubation periods,“**” represent the significance at the 0.01 level between two samples (*n* = 3).

To assess the efflux activity of RgMATE35, the RgMATE35-mCherry construct (Figure S2B of Supplementary File S2) and water (control) were introduced into *Xenopus oocytes*, respectively. In comparison to the control cells, the cells in which RgMATE35 was expressed and fused with the mCherry tag displayed red fluorescence at the plasma membrane (Figure 2A). This indicated that the RgMATE35 protein was heterogeneously expressed in the cell membrane of *X. oocytes*. Subsequently, the transport experiment of RgMATE35 was performed. The stable isotope-labeled [^2^H_3_]-FA and [^2^H_6_]-*p*CA injected into the oocyte cells were utilized as substrates, and the bathing medium (containing 96 mM NaCl, pH 7.5) from the extracellular environment served as the directed exchange cation. The HPLC-Q-TOF-MS method was utilized for analysis to quantify the efflux amount of the labeled FA and *p*CA exported into the bathing medium (with the inward Na^+^ ion gradient) from the expressing-RgMATE35 and control cells, respectively (Figure 2B,C, Table S3 of Supplementary File S1 and Figure S3 of Supplementary File S2). For example, following a 90 min incubation period, the efflux amounts of [^2^H_3_]-FA and [^2^H_6_]-*p*CA in the expressing-RgMATE35 cells were observed to be over 3-fold higher than those of the control. These results suggested that RgMATE35 is capable of exporting FA and *p*CA out of cells.

### 2.4. Functional Characterization of RgMATE35 in R. glutinosa

**Figure 3:**
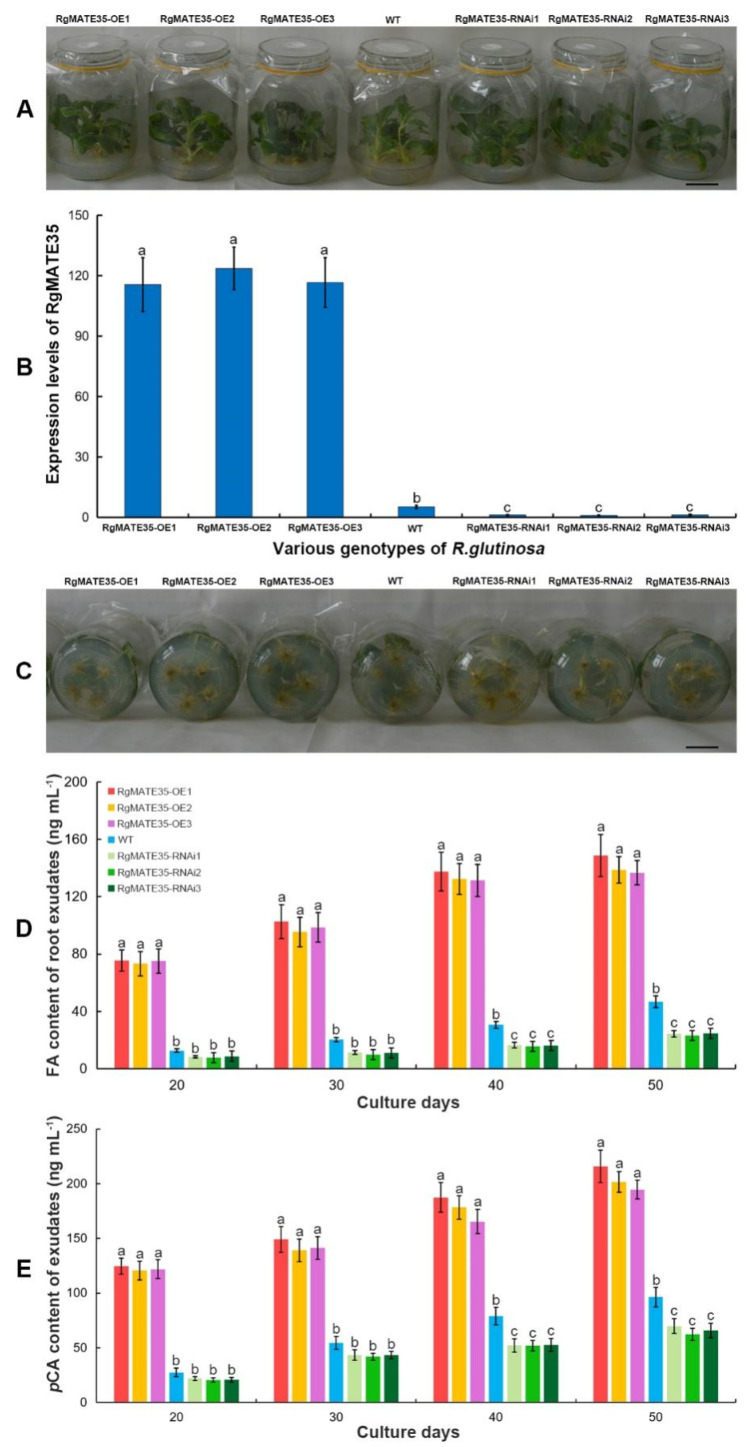
The expression profiles of the RgMATE35 and root-secreted phenolic acid profiles in the sterile seedlings of transgenic and WT *R. glutinosa*. (**A**) The seedlings after 30 days of cultivation; (**B**) the relative expression levels of *RgMATE35*; (**C**) the roots of the seedlings at 40 days; (**D**) the FA profile; (**E**) the *p*-CA profile. Scale bar = 4 cm; the error bars represent the standard error, and different lowercase letters indicate significance at the 0.05 level among the samples (*n* = 3).

To elucidate the molecular function of *RgMATE35* in vivo, we constructed the RgMATE35-OE and RgMATE35-RNAi vectors (Figure S2C,D of Supplementary File S2) with the CaMV35S promoter for overexpression and repression of the gene, respectively. The transgenic *R. glutinosa* lines overexpressing RgMATE35 (RgMATE35-OE) and those with knockdown RgMATE35 (RgMATE35-RNAi) were generated (Figure S4A–F of Supplementary File S2) and subsequently subjected to PCR and qRT-PCR analysis. The results demonstrated the specific amplification of a 385 bp fragment of the neomycin phosphotransferase II (*NPTII*) gene, which confers resistance to kanamycin, in the DNA of these transgenic lines, but not in that of the wild-type (WT) control (Figure S4G of Supplementary File S2). Furthermore, qRT-PCR analysis was performed in the roots of the various genotype *R. glutinosa* (Figure 3A,B, and Table S4 of Supplementary File S1), revealing that the expression levels of *RgMATE35* in the RgMATE35-OE lines were significantly elevated in comparison to the WT, whereas the levels in the RgMATE35-RNAi lines were significantly diminished in comparison to the WT.

To ascertain the function of RgMATE35 in the root secretion of phenolics in *R. glutinosa*, the FA and *p*CA contents of the root exudates in these transgenic sterile seedlings were analyzed (Figure 3C–E and Table S5 of Supplementary File S1). In comparison to the WT control, the RgMATE35-OE lines exhibited a notable increase in the contents of FA and *p*CA throughout the cultivation period, particularly after 40 days of cultivation (Figure 3D,E). For example, the FA and *p*CA contents from the RgMATE35-OE2 root exudates at the corresponding stages demonstrated a two-fold increase or more in comparison to those of the WT after 30 days of cultivation. In contrast, the RgMATE35-RNAi lines displayed a notable decline in these contents (Figure 3D,E) from the root exudates during the cultivation periods, particularly between 40 and 50 days. For example, the FA and *p*CA contents of the root exudates from the RgMATE35-RNAi3 lines after 40 days of cultivation were approximately 0.7-fold lower than those of the WT. Moreover, the same transgenic genotype lines (i.e., overexpression or RNAi lines) exhibited no significant differences in the release amounts throughout the cultivation periods. These findings indicated that *RgMATE35* overexpression promoted the secretion of FA and *p*CA in *R. glutinosa* roots, whereas *RgMATE35* repression had the opposite effect. Consequently, the RgMATE35-OE1 overexpression and RgMATE35-RNAi2 repression lines were selected for further experimentation.

### 2.5. Expression Pattern of RgMATE35 and Biomass Assessment in the Transgenic and WT R. glutinosa Under CM Stress

To ascertain whether RgMATE35 plays a regulatory role in the development of replant disease, the transgenic and WT *R. glutinosa* plants were subjected to CM stress. The CM stress treatments were applied to *R. glutinosa* plants, including those that were overexpressed and repressed *RgMATE35* (designated RgMAT35-OE1-CM and RgMAT35-RNAi2-CM, respectively), and wild-type plants (WT-CM). The plants were subsequently transplanted into pots containing soil that had been cultivated with *R. glutinosa* in the previous year. The normal growth (NG) WT plants (WT-NG, as a control) were transplanted into pots filled with soil that had not been grown with *R. glutinosa* for at least 10 years.

**Figure 4:**
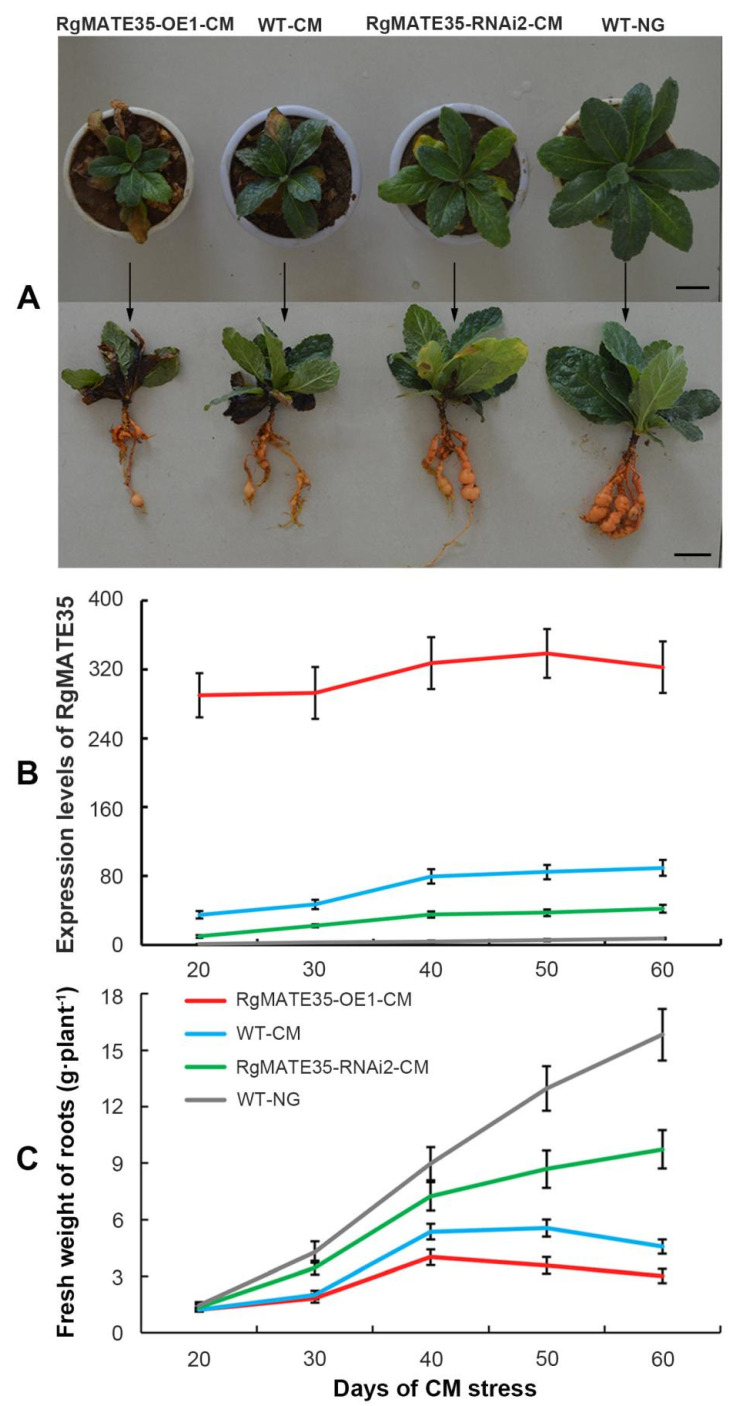
Expression profile of *RgMATE35* and root biomass assay in the transgenic and WT *R. glutinosa* subjected to CM stress over time. (**A**) Morphology from these plants at 60 days (scale bar = 3 cm); (**B**) expression profile; (**C**) fresh weight of roots.

The RgMATE35-OE1-CM, WT-CM, and RgMATE35-RNAi2-CM lines exhibited a range of inhibitory effects on plant growth and development (Figure 4A) when compared with the WT-NG plants. The expression profiles of *RgMATE35* in the three genotypes of *R. glutinosa* roots were quantified during the periods of CM stress (Figure 4B and Table S6 of Supplementary File S1). Overall, the expression levels of *RgMATE35* in the three *R. glutinosa* genotypes under CM stress at these stages (especially 50 and 60 days) were significantly higher than those of the WT-NG, indicating that CM stress resulted in the up-regulated expression of *RgMATE35* in *R. glutinosa*. Moreover, the levels of *RgMATE35* in the RgMATE35-OE1-CM plants were significantly elevated compared to those in the WT-CM between 30 and 60 days, whereas the levels of RgMATE35-RNAi2-CM plants exhibited a notable decline relative to those in the WT-CM. The results demonstrated that the overexpression and repression of *RgMATE35* resulted in an increase and decrease in its expression levels, respectively, in transgenic plants. This finding was consistent with the results obtained from the experiment illustrated in Figure 3B.

The biomass assessment revealed that CM stress resulted in a notable reduction in the biomass of *R. glutinosa* roots in these genotypes relative to the WT-NG plants at various stages. The most pronounced reduction was observed after 30 days of cultivation, with the lowest root weights evident in RgMATE35-OE1-CM, followed by WT-CM, and RgMATE35-RNAi2-CM (Figure 4C and Table S6 of Supplementary File S1). Moreover, the root weights of the overexpression plants (RgMATE35-OE1-CM) demonstrated a reduction of approximately 0.6-fold in comparison to WT-CM after 60 days of the stress. Conversely, the biomass of the roots from the repression plants (RgMATE35-RNAi2-CM) at the same stages exhibited an approximately 2-fold increase relative to that of WT-CM.

### 2.6. Physiological Characteristics of the Transgenic and WT R. glutinosa Under CM Stress

**Figure 5:**
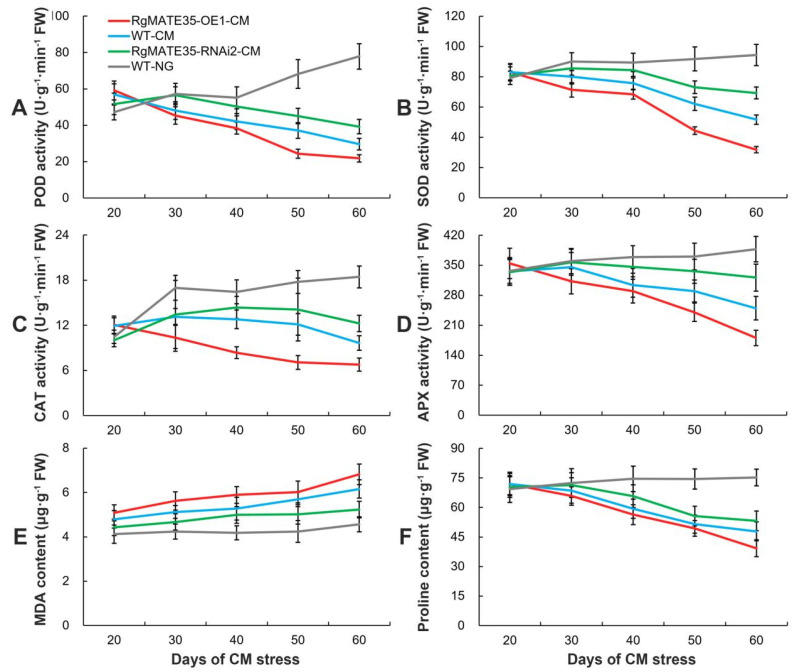
(**A**–**F**) Comparison of antioxidant enzyme activities, and the contents of MDA and proline in the transgenic and WT *R. glutinosa* roots subjected to CM stress and WT under normal conditions over time.

To assess the extent of injury in different genotypes of plants subjected to CM stress, the activities of antioxidant enzymes, including peroxidase (POD) and superoxide dismutase (SOD), catalase (CAT) and ascorbate peroxidase isozyme (APX), were examined. Moreover, the levels of malondialdehyde (MDA) and proline were quantified in the roots of *R. glutinosa* (Figure 5 and Table S7 of Supplementary File S1). The results demonstrated that the activities of these antioxidant enzymes in the three genotype roots under CM stress between 30 and 60 days were significantly increased in comparison to those of WT-NG roots (Figure 5A–D). Furthermore, the activities of RgMATE35-OE1-CM roots were observed to be significantly lower than those of WT-CM roots, whereas the activities of RgMATE35-RNAi2-CM roots were significantly higher than those of WT-CM roots among the diverse genotype roots subjected to CM stress. Similarly, the MDA content exhibited a comparable trend. After a period of 30 to 60 days of CM stress, the order of the roots was as follows: RgMATE35-OE1-CM > WT-CM > RgMATE35-RNAi2-CM > WT-NG roots (Figure 5E). Conversely, the proline contents exhibited an inverse pattern: RgMATE35-OE1-CM < WT-CM < RgMATE35-RNAi2-CM < WT-NG roots (Figure 5F).

**Figure 6:**
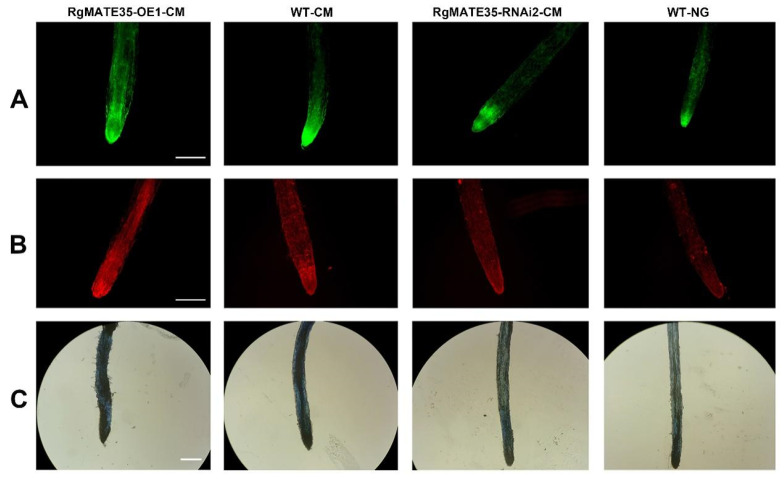
Detection of ROS and cell death in the root tips of transgenic and WT plants under CM stress at 60 days. (**A**) DCFH-DA staining; (**B**) PI staining; (**C**) trypan blue staining. Scale bar = 320 μm.

The accumulation of reactive oxygen species (ROS) and subsequent cell death was observed in the root tips at 60 days after CM stress through staining analysis (Figure 6). Overall, the levels of ROS in the root tips of the various genotypes of *R. glutinosa* were significantly higher under CM stress than in WT-NG roots. Furthermore, the accumulation of ROS in RgMATE35-OE1-CM roots was significantly higher than that in WT-CM roots, with the entire zone of the root tips stained with 2,7-dichlorofluorescein diacetate (DCFH-DA) exhibiting elevated ROS levels. The accumulation in RgMATE35-RNAi2-CM roots was comparatively lower than that in WT-CM roots (Figure 6A). To identify the stress-induced cell death in root tips, the roots were stained with propidium iodide (PI) and trypan blue dyes. As shown in Figure 6B,C, the WT-NG plants displayed minimal to no staining. However, distinct patterns of stained cells were observed in the root tips from the three genotype plants subjected to CM stress. In particular, the regions stained in the roots of these plants under CM stress were notably expanded, resulting in an obvious increase in the number of PI- and trypan-blue-positive cells and a heightened staining intensity when compared with the WT-NG roots. It is noteworthy that the root tips of RgMATE35-OE1-CM plants exhibited the most pronounced staining among the samples.

## 3. Discussion

Phenolics are secreted from plant roots into the soil, which represents a key factor that leads to autotoxicity by directly or indirectly affecting the rhizosphere environment in plants subjected to CM stress [1,2,3,4]. It is therefore crucial to elucidate the transport mechanisms of plant allelopathic phenolics in order to address the challenges posed by replant disease. RgMATE35, a member of the MATE protein family, has been proposed to function as an efflux transporter for phenolics [31]. To test this hypothesis, we examined the tissue-specific expression patterns of *RgMATE35* in *R. glutinosa*, which exhibited the highest expression levels at the roots, particularly the root tips. As has been previously documented, several well-characterized *MATE* genes involved in the efflux of metabolites often exhibit tissue-specific transcript patterns in plants, especially with higher expression observed in the root apical regions where the efflux of some metabolites typically occurs [26,27,30]. The expression pattern of *RgMATE35* was similar to that observed in other reported MATE transporters from *O. sativa* [27,28], *C. melo* [30], *Hordeum vulgare* [34] and *G. max* [20]. This suggested that *RgMATE35* may be involved in the efflux of phenolics from plant roots. Additionally, the expression levels of *RgMATE35* in *R. glutinosa* between 60 and 90 days, when the secretion of phenolics from the plant roots was particularly robust [35,36], were significantly higher compared to other growth stages. These findings thus suggested that the elevated expression of *RgMATE35* during these stages could facilitate the transport of phenolics. Furthermore, the transient co-expression analysis corroborated the plasma membrane localization of RgMATE35, aligning with the localization patterns observed in other plant MATE transporters, including those from *A. thaliana* [37], *G. max* [20] and *S. bicolor* [29]. Therefore, it was postulated that RgMATE35 would exhibit the typical characteristics of plant efflux transporters. It has been demonstrated that plant MATE efflux transporters, such as *Zea mays* ZmMATE1, have been demonstrated to mediate organic anion efflux coupled with cation influx, including Na^+^ influx [19,20,23,38]. In this study, the assessment of transport activity in *X. oocytes* expressing *RgMATE35* revealed that it functions as an antiporter, exporting FA and *p*CA out of cells across the membrane. This suggested that RgMATE35 possesses the ability to efflux phenolics.

Previous research has demonstrated a positive correlation between the transcript abundances of *MATEs* and the transport amounts of their corresponding substrates in plants [27,28,29,30,37,38,39]. In particular, *A. thaliana AtDTX18*, which enables the export of coumaroylagmatine from cells, is highly expressed, thereby enhancing the secretion of coumaroylagmatine in plants, whereas the deletion of this gene resulted in the repression of the extracellular export of coumaroylagmatine [24]. Moreover, the mutation of *AtDTX50*, which encodes an ABA efflux transporter protein in *A. thaliana*, resulted in a significant reduction in ABA release from cells [19]. The overexpression of *OsPEZ1* and *OsPEZ2*, which are involved in the efflux of phenolics, resulted in an increased secretion of phenolic acids in rice roots, while the mutation in these genes led to a reduction in the secretion amounts [27,28]. Furthermore, the knockdown of *CmMATE1*, which encodes a MATE transporter involved in the extrusion of cucurbitacins, notably decreased the secretion level of these compounds in melon [30]. In this study, the overexpression of *RgMATE35* was observed to significantly enhance the secretion of FA and *p*CA from *R. glutinosa* roots, whereas the repression of *RgMATE35* resulted in a significant reduction in the secretion of these phenolic acids. These findings indicated that RgMATE35 plays a positive role in the secretion of phenolic acids from plant roots into the rhizosphere.

The excessive accumulation of phenolic allelochemicals, whether as a result of direct harm or indirect microbial mediation, can result in autotoxicity in plants subjected to CM stress [2,9,13,40]. It is well established that the autotoxic injury of allelopathic phenolics plays a pivotal role in the development of replant disease in *R. glutinosa* and other plant species [9,41,42,43,44]. The MATE/DTX family has been previously documented to play a role in plant growth and development, various stress responses and other biological processes by controlling the secretion of some metabolites (including phenolics) [24,37,39]. In *Arachis hypogaea*, the citrate transporter gene *AhFRDL1* was found to be highly transcribed in root tips subjected to Al stress, indicating that it contributes to Al tolerance by promoting citrate exudation [45]. In *A. thaliana*, *AtDTX18*, which is involved in the transport of coumaroylagmatine, is strongly expressed when leaves are inoculated with *Phytophthora infestans*. This suggested that the pathogen’s infection is inhibited by the enhancement in coumaroylagmatine secretion [24]. The findings of this study demonstrated that RgMATE35 plays a role in the efflux of allelopathic phenolic acids. Moreover, the expression of *RgMATE35* in the roots of *R. glutinosa* (across various genotypes) was markedly induced by CM stress. It was therefore hypothesized that the higher expression of *RgMATE35* may accelerate the development of replant disease by increasing the root secretion of phenolic acids.

The phenomenon of replant disease is characterized by several key factors, including a reduction in biomass, diminished antioxidant capabilities, elevated levels of reactive oxygen species (ROS), and subsequent cell death [2,41]. The data presented in this study demonstrated that both the *RgMATE35* transgenic plants and the WT plants subjected to CM stress exhibited a significant reduction in root biomass. Furthermore, the plants displayed a decline in the activities of root antioxidant enzymes, accompanied by a notable increase in the accumulation of ROS and an elevation in cell death within the root tips. The injury effects were markedly more pronounced in the plants subjected to CM stress compared to the WT plants grown under normal growth conditions. It is noteworthy that the *RgMATE35* overexpression plants subjected to CM stress exhibited the most severe injury. In contrast, the plants in which *RgMATE35* was repressed under the stress exhibited the least injury. It has been elucidated that the degree of autotoxic injury correlates positively with the abundance of allelochemicals present in the plant rhizosphere [43,44,46]. The results of this study demonstrated that RgMATE35 functions as a transporter that facilitates the export of allelopathic phenolic acids into the plant rhizosphere. The overexpression of *RgMATE35* in *R. glutinosa* subjected to CM stress may facilitate the secretion and accumulation of these phenolic acids in the plant rhizosphere, resulting in a more severe injury and strengthening the development of replant disease. However, the repression of *RgMATE35* resulted in a reduction in the root secretion and rhizosphere accumulation of these phenolic acids, which mitigated the injury to plants and provided a slight alleviation of the disease. Furthermore, the repression of the gene may facilitate the accumulation of these phenolic acids (also as active compounds) within plant cells, potentially enhancing the plant resistance to various stresses, as previously described [47]. Therefore, RgMATE35, which functions as an efflux transporter, positively regulates the development of replant disease in *R. glutinosa* by facilitating the root secretion of phenolic acids.

In summary, this study provides a theoretical framework for elucidating the underlying mechanism of allelochemicals’ export to the rhizosphere in the context of plant replant disease development. In addition to phenolic compounds, plants secrete a diverse array of other allelopathic substances, including glycosides and terpenoids [4,36,44]. However, the functional characteristics of numerous plant MATEs involved in the efflux of allelochemicals remain poor. Further research is necessary to investigate additional MATE transporters responsible for the efflux of allelochemicals, which will enhance our understanding of the regulatory mechanisms implicated in the progression of plant replant disease. It is hypothesized that the disruption of key *MATE* genes, such as *RgMATE35*, may result in a reduction in the efflux capacity of allelochemicals, thereby reducing the autotoxic injury to the plants and ultimately relieving replant disease. Further investigation into additional efflux transporters may reveal that plant transporter resources can be more effectively utilized to develop new techniques and targeted strategies for controlling and mitigating diseases.

## 4. Materials and Methods

### 4.1. R. glutinosa Culture, Total RNA Isolation and Reverse Transcription

The *R. glutinosa* cultivar “Wen 85-5” was cultivated in pots filled with soil, which were subsequently analyzed for a range of chemical properties. The chemical properties of the soil included the following: organic matter (10.04 g kg^−1^), total nitrogen (2.46 g kg^−1^), total phosphorus (0.51g kg^−1^), total potassium (1.14 g kg^−1^), hydrolysable nitrogen (21.63 mg kg^−1^), phosphorus (34.27 mg kg^−1^) and potassium (305.76 mg kg^−1^). The soils exhibited a pH value and a cation exchange capacity of 133.7 mmol kg^−1^. It had not been utilized for the cultivation of this species for at least 10 years. The plants were grown in a greenhouse with a constant temperature of 26 °C and a 14-h/10 h (light/dark) photoperiod, with humidity maintained at 65%. The experiment was conducted at the School of Bioengineering, Henan University of Technology (Zhengzhou, Henan, China). The plants were cultivated between 1 April and 31 December 2023. The soil in each pot was irrigated with 300 mL of tap water every 5–7 days. To analyze the expression pattern and clone of *RgMATE35*, a range of tissues (including root tips, fibrous roots, tuberous roots, stems, old leaves, and functional leaves) were collected at various cultivation stages (including 30 days after planting (seedlings), 60 days (root elongation), 90, 120 and 150 days (early, middle and late tuberous root expansion)) from three biological replicates in the preliminary observations.

To facilitate the isolation of total RNA, each sample was frozen in liquid nitrogen. Total RNA was extracted from each sample using TRIzol™ reagent (Invitrogen, Carlsbad, CA, USA), following the manufacturer’s instructions. The integrity and purity of the total RNA were assessed using the 2100 Bioanalyzer (Agilent Technologies, Inc., Santa Clara, CA, USA) and quantified with the NanoDrop™ 2000 (Thermo Scientific, Wilmington, DE, USA), respectively [32,33]. A 1 μg aliquot of total RNA from each sample was reverse-transcribed into cDNA using HiScript^®^ III Reverse Transcriptase (Vazyme, Nanjing, China) and oligo-dT primers by the manufacturer’s instructions. The obtained cDNA samples were further diluted to a 1:5 ratio in subsequent experiments.

### 4.2. QRT-PCR

To determine the expression levels, the primers for *RgMATE35* (GenBank ID: MK120947.1) were designed using the Beacon Designer™ 8.0 software (Table S8 of Supplementary File S1). QRT-PCR analysis was conducted using SYBR Green PCR Master Mix (2×) (Fermentas, Canada Co., Ltd., Burlington, ON, Canada) on a BIO-RAD iQ5 real-time PCR detection system (Bio-Rad Laboratories, Inc., Hercules, CA, USA), as previously described [48]. Briefly, each 50 μL PCR reaction consisted of 1 μL cDNA, 1 μL 1 μM forward primer, 1 μL 1 μM reverse primer, 25 μL SYBR Green PCR Master Mix and 22 μL nuclease-free water. The thermocycler settings were as follows: The initial denaturation was conducted at 95 °C for 2 min, followed by 38 cycles of denaturation at 95 °C for 10 s, annealing at 58 °C for 20 s, and extension at 72 °C for 15 s. In addition, the melting curves were generated at temperatures ranging from 55 °C to 95 °C to confirm the specificity of the reaction. Each sample was assessed with three biological and technical replicates. The relative expression levels of *RgMATE35* were normalized using the *RgActin* reference gene (Genbank ID: EU526396.1), and the 2^−ΔΔCT^ method was employed to calculate the data, as previously described by Livak and Schmittgen [49].

### 4.3. Construction of Various Expression Vectors

The complete and partial open reading frame (ORF) sequences of *RgMATE35* were amplified by PCR using PrimeSTAR^®^ HS DNA Polymerase (Takara, Tokyo, Japan) and primer sets listed in Table S8 of Supplementary File S1 for different expression vectors. To determine the subcellular localization, the *RgMATE35* ORF sequence lacking the stop codon was inserted into a pBI121 vector (*Kpn*I digestion site) (Miaoling Biology, Wuhan, China) with a CaMV35S promoter, fused with the C-terminal region of the *GFP* gene, to generate the CaMV35S:RgMATE35-GFP construct (Figure S2A of Supplementary File S2). To express *RgMATE35* in *X. oocytes*, the complete ORF was cloned into a pGEMHE-mCherry vector (*Bgl*II and *Xba*I digestion sites) under the control of the T7 promoter (Miaoling Biology, Wuhan, China), resulting in the RgMATE35-mCherry vector (Figure S2B of Supplementary File S2). To overexpress *RgMATE35* in *R. glutinosa*, the complete ORF was cloned into a pBI121 vector with a CaMV35S promoter containing a fragment of the *NPTII* gene, which confers resistance to kanamycin. This resulted in the RgMATE35-OE construct, which is shown in Figure S2C of Supplementary File S2. To repress the *RgMATE35* transcript, the partial ORF fragment of *RgMATE35* was cloned into the pRNAi-GG vector (Biovector Science Lab, China) with a CaMV35S promoter and the *NPTII* gene, resulting in the RgMATE35-RNAi repression construct (Figure S2D of Supplementary File S2). The resulting constructs were then transformed into *Escherichia coli* strain DH5α, and the positive clones were verified by sequencing (Sangon, Shanghai, China).

### 4.4. Subcellular Localization Determination

Mesophyll protoplasts were isolated from the leaves of *R. glutinosa* at the six-leaf stage using the modified version of the *A. thaliana* mesophyll protoplast procedure [50]. In brief, 1 g of the well-expanded leaves from the 30-day-old sterile seedlings (usually leaf numbers four to six) was selected and approximately 1 mm leaf strips were excised from the midrib of a leaf using a sharp blade. The leaf strips were immersed in 10 mL enzyme storage solution containing 0.5 M mannitol, 20 mM MES (pH 5.8), 20 mM KCl and 5 mM CaCl_2_ for 2 h at room temperature and in the dark. Subsequently, the leaf strips were filtered using a 200 mesh nylon filter and digested in 10 mL of enzyme storage solution containing 2.0% (wt/vol) cellulase R10 (Yakult, Tokyo, Japan) and 0.6% (wt/vol) pectolase Y-23 (Yakult, Tokyo, Japan) for about 5 h with a gentle swirling motion (100 rpm) at room temperature and in the dark. The digested solution was filtered using a 200-mesh nylon filter and centrifuged at 1200× *g* for 5 min; the precipitation was resuspended with 10 mL enzyme storage solution containing 21% (wt/vol) and centrifuged at 1200× *g* for 3 min. The top and bottom layers of the solution were removed, and the protoplast band at the middle layer was transferred to a new 10 mL tube. It was then diluted by supplying 5 mL of enzyme storage solution and centrifuged at 100× *g* for 3 min. It is important to remove as much of the supernatant as possible and to resuspend the protoplast pellet by adding 1 mL of enzyme storage solution and gently swirling. A 10 μL protoplast suspension solution was transferred to test its density by an optical microscope. The remaining protoplast was prepared for the subsequent transformation.

The *RgMATE35* and *NAA60* genes were fused to GFP and mKate, respectively. Approximately 10 μg of the plasmids for CaMV35S:RgMATE35-GFP or CaMV35S-GFP, which was fused to GFP, and CaMV35S:NAA60-mKate, which was mKate (Biorun BioSciences, Wuhan China), was transiently co-transformed into protoplasts by the polyethylene glycol-mediated method [50]. Following a 36–48 h incubation period at 25 °C, fluorescence in the transformed protoplasts was observed using a Leica SP8 confocal microscope (Leica, Wetzlar, Germany). Excitation of the GFP and mKate was performed at 488 and 561 nm, respectively, with emission signals detected using 507 and 580 nm band-pass filters. Each experiment was performed in triplicate.

### 4.5. Heterologous Expression and Transport Activity Assay of RgMATE35 in X. oocytes

For mRNA transcription in *X. oocytes*, the RgMATE35-mCherry construct was linearized using *Nhe*I, and cRNA was synthesized using the mMESSAGE mMACHINE™ T7 Transcription Kit (Invitrogen) according to the manufacturer’s instructions. A total volume of 30 nL containing 60 ng of each cRNA or water (serving as a control) was injected into each oocyte. Subsequently, the oocytes were incubated at 16 °C in ND96 buffer (96 mM NaCl, 5 mM HEPES, 2 mM KCl, 1 mM CaCl_2_, 1 mM MgCl_2_ and 50 mg L^−1^ ampicillin (adjusted to 7.5 pH with NaOH)) for 36 h. To detect heterologous expression, three randomly selected cells were observed using a Leica SP8 confocal microscope. Excitation of mCherry was performed at 587 nm, and the emitted signal was detected using a 610 nm band-pass filter. For the export assay, each oocyte was injected with 30 nl of 500 µM stable isotope-labeled [^2^H_3_]-FA or [^2^H_6_]-*p*CA (IsoReag, Shanghai, China), and then placed in ND96 buffer and kept on ice for five minutes. Each sample of five oocytes was incubated in 100 μL of ND96 buffer for 0–120 min at 16 °C. After, the buffer was collected and extracted with an equal volume of 80% methanol/water (v/v). The extracted solutions were filtered through a 0.22-µm filter in preparation for HPLC-Q-TOF-MS analysis.

The evaluation of [^2^H_3_]-FA or [^2^H_6_]-*p*CA was conducted via HPLC-Q-TOF-MS using a 1200 HPLC system (C18 column (2.1 × 150 mm, 3.5 μm)) coupled to an Agilent Q-TOF 6520 mass spectrometer (Agilent, Santa Clara, CA, USA) equipped with an electrospray ionization (ESI) device. The mobile phase employed for each sample was a 65:35 mixture of 0.1% acetic acid and 100% acetonitrile with a constant flow rate of 1 mL min^−1^. To enhance the specificity of the analysis, the multiple reaction monitoring (NRM) scan type was employed in negative scan mode. The detection of [^2^H_3_]-FA or [^2^H_6_]-*p*CA was determined by monitoring the increase in maximum absorbance at wavelengths of 311 and 254 nm, respectively. The experiment was performed in triplicate.

### 4.6. Generation of the Transgenic Lines of R. glutinosa

The RgMATE35-OE and RgMATE35-RNAi constructs were, respectively, transformed into the *Agrobacterium tumefaciens* strain GV3101 strain using the freeze–thaw method, as previously described [51]. For the overexpression and repression of *RgMATE35*, the sterilized leaves of *R. glutinosa* seedlings were subjected to *Agrobacterium*-mediated transformation, and transgenic seedlings were generated and cultured as previously described [52]. In brief, the 50 mL bacterial suspensions (OD_600_ = 0.8) of *A. tumefaciens* harboring the RgMATE35-OE or RgMATE35-RNAi transformation constructs were subjected to centrifugation, whereby the supernatant was removed and the remaining precipitation of *A. tumefaciens* was resuspended (OD_600_ = 0.8) in callus induction liquid medium containing 4.3 g L^−1^. The solidified MS nutrient medium (PhytoTech Labs, Lenexa, KS, USA) was supplemented with 30 g L^−1^ sucrose, 0.5 mg L^−1^ NAA, 3 mg L^−1^ 6-BA and 100 mg·L^−1^ kanamycin, 200 100 mg L^−1^ acetosyringone, at pH 5.8. The mature leaves of 40-day-old sterile *R. glutinosa* seedlings were sliced into 5 mm^2^ pieces, and approximately 300 leaf piece explants were immersed in the callus induction MS suspension. Subsequently, the explants were incubated for 30 min with shaking at 100 rpm, after which they were blotted dry with autoclaved filter paper. The explants with the *A. tumefaciens* were subjected to co-cultivation in the induction solidified medium and incubated for a period of 48 h at 26 °C. The explants were transferred to callus induction/selection solidified medium containing 4.3 g L^−1^ MS medium and 30 g L^−1^ sucrose, 0.5 mg L^−1^ NAA, 1 mg L^−1^ 6-BA, 100 mg L^−1^ kanamycin, 200 mg L^−1^ timentin, and 7.8 g L^−1^ agarose at a pH of 5.8. The explants were transferred to a fresh medium at three-week intervals. Once adventitious buds had developed from the calli to a height of approximately 3 cm in height, they were dissected and transferred to a new culture bottle containing shoot elongation medium consisting of MS medium, 30 g L^−1^ sucrose, 0.5 mg L^−1^ NAA, 3 mg L^−1^, 100 mg L^−1^ kanamycin, 200 mg L^−1^ timentin, and 7.8 g L^−1^ agarose. The resulting shoots, which had formed a few leaves, were transferred to a rooting medium containing 4.3 g L^−1^ MS nutrient medium, 30 g L^−1^ sucrose, 0.05 mg L^−1^ NAA, 100 mg L^−1^ kanamycin and 7.8 g L^−1^ agarose. To determine whether the overexpression or repression construct had been integrated into the genomes of these transgenic plants, the transgenic and WT lines were cultivated in a sterile MS culture medium (Solarbio, Beijing, China) for 30 days in the greenhouse as mentioned above. Genomic DNA was extracted from the leaves of the transgenic lines using the cetyltrimethylammonium bromide (CTAB) method [53]. The *NPTII* gene was amplified from the extracted DNA samples, and the transgenic lines with positive results were identified. The expression level of *RgMATE35* in the positive transgenic lines was determined using the qRT-PCR method described above. Each sample contained three biological replicates.

### 4.7. Determination of FA and pCA Contents from Root Exudates of R. glutinosa Seedlings

Transgenic and WT *R. glutinosa* seedlings were subcultured with 40 mL of the MS medium under the aforementioned sterilized conditions. The root exudates were collected from the medium at 20, 30, 40, and 50 days, respectively. To estimate the FA and *p*CA contents of the root exudates, the mediums were collected, 5 mL of 1M HCL was added, the samples were heated at 60 °C, the solutions were filtered, and the filtrates were extracted three times in an equal volume of ethyl acetate. The pooled extracts were lyophilized, and the residue was dissolved in 2 mL of methanol. This solution was filtered through a 0.22 µm filter and quantified for the measurement of FA and *p*CA contents by HPLC analysis, as described above.

### 4.8. Culture and Collection of R. glutinosa Subjected to CM Stress

To induce CM stress in *R. glutinosa*, the transgenic and WT seedlings were initially transplanted at the six-leaf stage into pots containing an organic-matrix-based nutrient medium and then grown for approximately 20 days (i.e., from 1 to 21 March 2024) (Figure S4H of Supplementary File S2). Subsequently, for CM stress treatment, the transgenic *R. glutinosa* (RgMATE35-OE1 and RgMATE35-RNAi2), and the WT plants (a positive control) were transplanted into pots filled with soil that had been used to grow with *R. glutinosa* plants between 1 April and 31 December 2023 and then cultured from 22 March to 22 June 2024. During this period, the WT plants (non-stress, a negative control) were also transplanted into pots filled with soil (which had not been used to grow *R. glutinosa plants* for 10 years) from 22 March to 22 June 2024 (Figure S4I of Supplementary File S2). The plants were cultivated in the aforementioned greenhouse. Following a period of 20, 30, 40, 50, and 60 days, the roots from these plants were harvested for further analysis.

### 4.9. Measurement of Molecular and Physio-Biochemical Characteristics of R. glutinosa

The expression levels of *RgMATE35* from these roots were assessed by the qRT-PCR method, as mentioned above. The fresh roots were analyzed for the activities of their POD [54], SOD [55], CAT [56], and APX [57], and the contents of MDA [58] and proline [55]. Each experiment was performed with three biological replicates.

The detection of ROS in the root tips of 60-day-old plants was performed using the DCFH-DA staining method, as described by Duan et al. [59]. Detection of cell death was performed using PI and trypan blue staining, as previously described [59]. The stained root tips were then visualized by fluorescence or light microscopy (Axioskop; Carl Zeiss, Jena, Germany). Each experiment was repeated at least three times.

### 4.10. Statistical Analysis

All data were subjected to analysis using the statistical software package SPSS 20.0. The error bars displayed the standard deviation (SD) of the mean, derived from three independent replicates. The different levels of genes and other biochemical indices in *R. glutinosa* were evaluated using one-way analysis of variance (ANOVA), with subsequent multiple comparisons conducted using Fisher’s least significant difference (LSD) test (*n* = 3, *p* < 0.05). Student’s *t*-test of independent samples was employed to evaluate the differences in the efflux abundances of phenolic acids between the expressing-*RgMATE35* and control *X. oocytes* (*n* = 3, *p* < 0.01).

## 5. Conclusions

This study presents the first report of the transport activity and functional characterization of a MATE-type transporter, RgMATE35, derived from *R. glutinosa*. The analysis of the gene expression pattern revealed that it is robustly expressed in root tissues. The subcellular localization of RgMATE35 was determined to be plasma membranes. The assessment of transport activity in *X*. *oocytes* demonstrated that RgMATE35 facilitated the efflux of two allelopathic phenolics, FA and *p*CA, from the cells. The overexpression and repression function experiments in *R. glutinosa* confirmed that RgMATE35 is involved in the secretion of FA and *p*CA. The assessment of autotoxic injury by the molecular and physiological characteristics of the transgenic *R. glutinosa* subjected to CM stress revealed that RgMATE35 positively modulates the development of plant replant disease through the root secretion of phenolic acids. Further research is required to investigate more MATE transporters involved in the efflux of allelochemicals. This will improve our understanding of the regulatory mechanisms in the development of plant replant disease; the findings establish a fundamental framework for elucidating the molecular regulatory mechanism through which MATEs regulate replant disease through the secretion of allelochemicals from plant roots into the rhizosphere. This will facilitate the development of precise and efficient strategies to alleviate this significant agricultural problem.

## Data Availability

Data are contained within the article.

## Supplementary Materials

The following supporting information can be downloaded at: https://www.mdpi.com/article/10.3390/plants13213007/s1, Table S1: Quality assay of isolated RNAs in various tissues of *R. glutinosa* during these cultivation periods; Table S2: The *RgMATE35* expression profiles in various tissues of *R. glutinosa* during these cultivation periods; Table S3: The [^2^H_3_]-FA and [^2^H_6_]-*p*CA efflux amounts (μM) was tested by RgMATE35 transport during these incubation periods; Table S4: Statistical data of the *RgMATE35* expression levels from transgenic *R. glutinosa* seedlings; Table S5: Statistical data of root-secrected phenolic acid amounts in the transgenic and WT *R. glutinosa* sterile seedlings; Table S6: Statistical analysis of expression levels of *RgMATE35* and root biomass in the transgenic and WT *R. glutinosa* subjected to CM stress over time; Table S7: Oxidative stress indices in the roots of the transgenic *RgMATE35* and WT plants subjected to CM stress; Table S8: The primer sequences of *RgMATE35* used to clone and constructs, and the primer sequences of positive screened gene in *R. glutinosa*; Figure S1: A sample data for the RNA qualify from the functional leaves of *R. glutinosa* at 30 days of cultivation using Agilent 2100 analyzer. Figure S2: The constructs for various *RgMATE35* vectors; Figure S3: HPLC-Q-TOF-MS profiles of the labeled [^2^H_3_]-FA and [^2^H_6_]-pCA efflux amounts in expressing-*RgMATE35* and control *X. oocytes* at the 90-minute time point. Figure S4: Generation of transgenic *RgMATE35 R. glutinosa* plants.
