# Supplementary material for: Rehmannia glutinosa RgMATE35 Participates in the Root Secretion of Phenolic Acids and Modulates the Development of Plant Replant Disease
Source: Plants (Basel). 2024 Oct 28;13(21):3007. doi: 10.3390/plants13213007 (PMC11548660; doi:10.3390/plants13213007)
Supplement: Supplementary file 1 [file plants-13-03007-s001.zip › Supplementary File S2.pdf]

Supplementary File S2 includes 4 subfigures

Figure S1 A sample data for the RNA qualify from the functional leaves of *R. glutinosa* at 30 days of cultivation using Agilent 2100 analyzer.

.

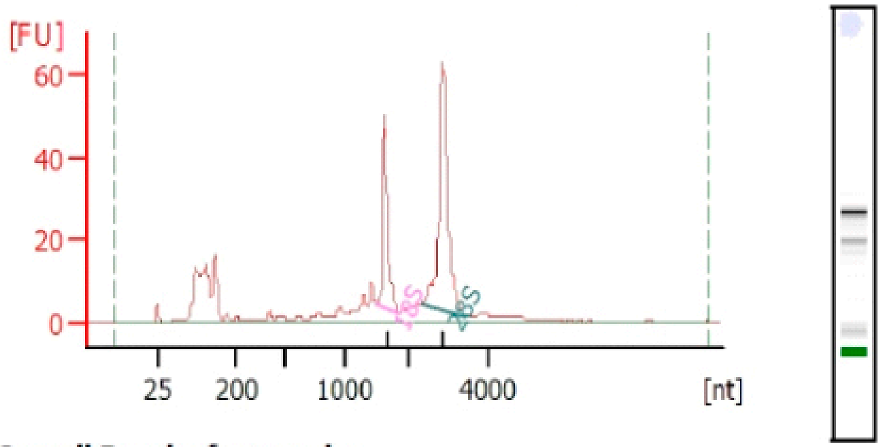

Overall Results for sample :

RNA Area:	400.3
RNA Concentration:	416 ng/µl
rRNA Ratio [28s / 18s]:	2.1
RNA Integrity Number (RIN):	8.6 (B.02.07)
Result Flagging Color:	
Result Flagging Label:	RIN: 8.60

Fragment table for sample :

Name	Start Size [nt]	End Size [nt]	Area	% of total Area
18S	1,497	1,842	54.2	13.5
28S	2,361	3,441	111.8	27.9

Figure S2 The constructs for various RgMATE35 vectors. These include: (A) subcellular localization (CaMV35S:RgMATE35-GFP), (B) heterogeneous expression (RgMATE35-mCherry), (C) overexpression (RgMATE35-OE), and (D) RNAi repression (RgMATE35-RNAi).

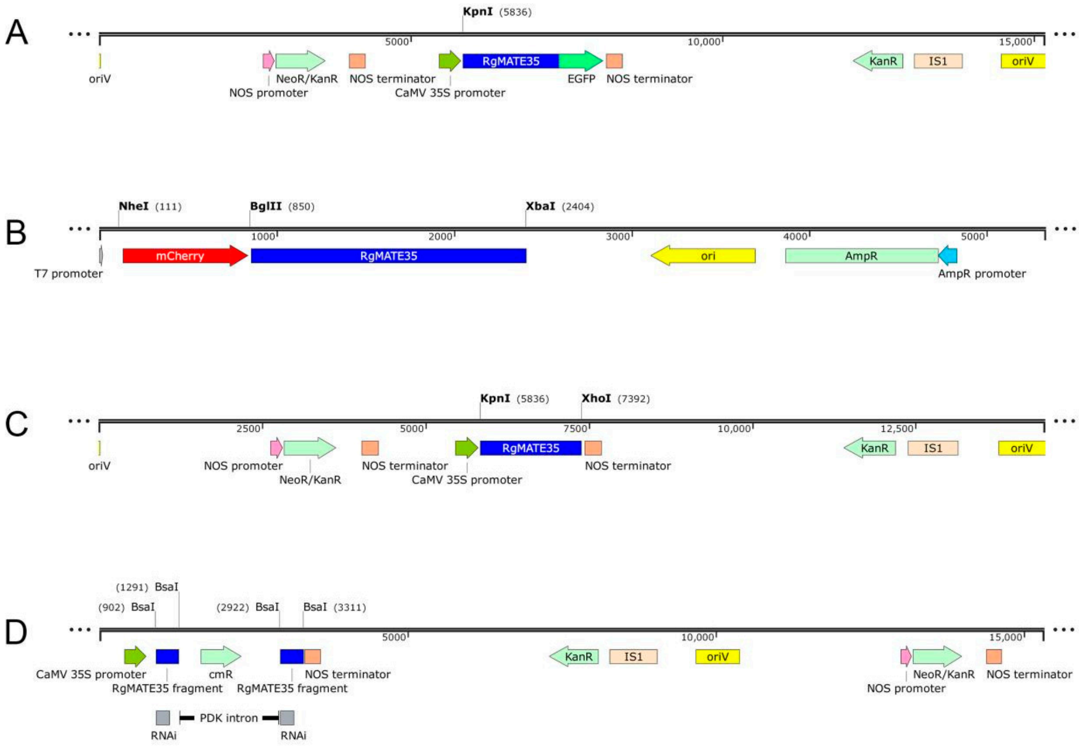

Figure S3 HPLC-Q-TOF-MS profiles of the labeled [$^2H_3$]-FA and [$^2H_6$]-*p*CA efflux amounts in expressing-RgMATE35 and control *X. oocytes* at the 90-minute time point. The mass (A) and HPLC (B) spectra of the labeled phenolic acid standards are presented, along with the HPLC spectra of efflux phenolic acids from expressing-RgMATE35 (C) and control (D) cells.

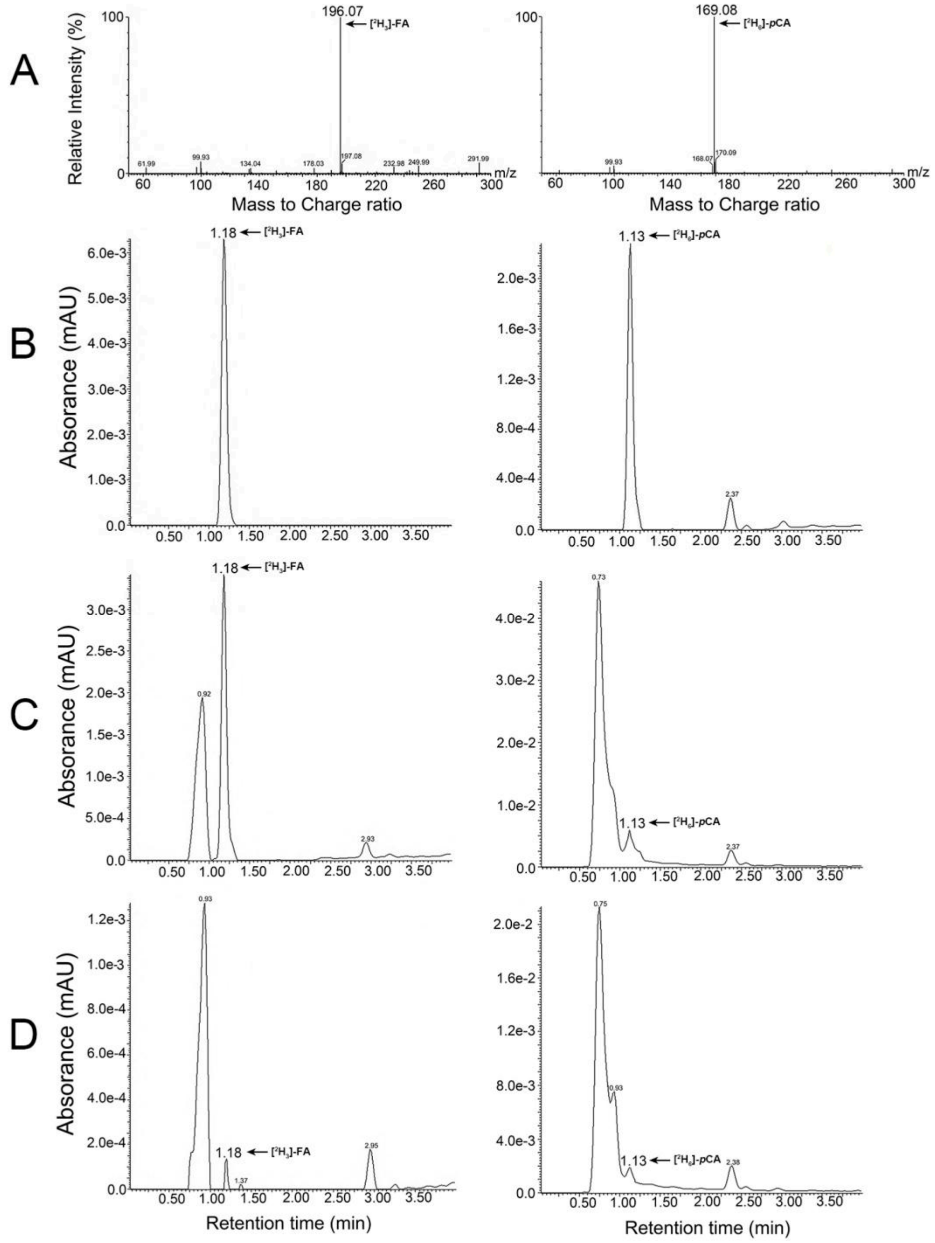

Figure S4 Generation of transgenic *RgMATE35 R. glutinosa* plants. (A) Co-cultivation of the explants and *Agrobacterium tumefaciens*; (B) and (C) the callus induction/selection medium from the explants-transferred; (D) the adventitious buds induction/selection medium from the callus; (E) and (F) the transgenic seedlings were generated in the selection medium; (G) agarose gel images of *NPTII* PCR products from the positive transgenic *RgMATE35* and wild-type (WT) plants; (H) these seedlings-transplanted to pots in organic matrix nutrition soils to facilitate adaption to soil environments; (I) these seedlings were transplanted to pots in field soils. Scale bar = 4 cm.

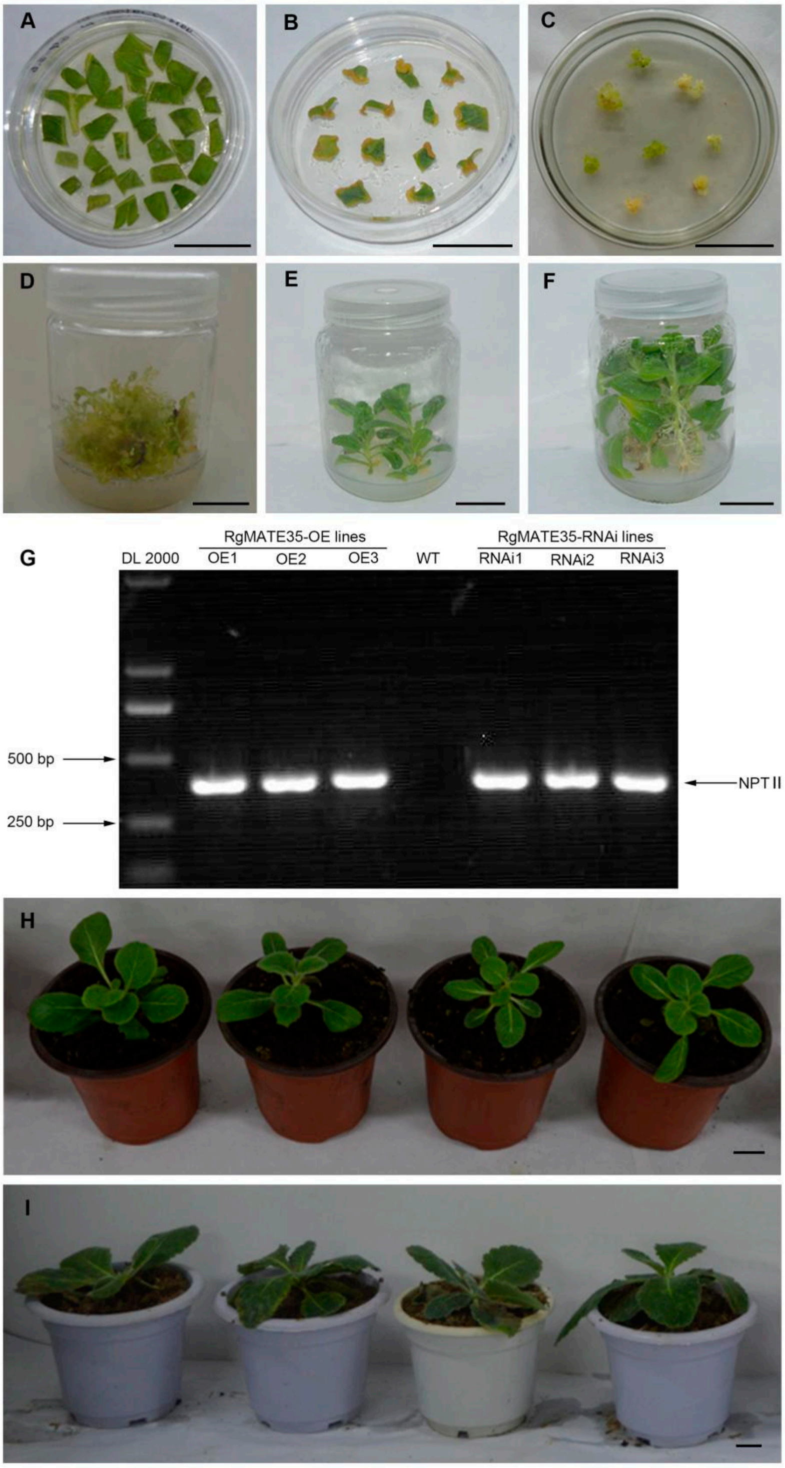
A
B
C
D
E
F
G
DL 2000
RgMATE35-OE lines
OE1
OE2
OE3
WT
RgMATE35-RNAi lines
RNAi1
RNAi2
RNAi3
500 bp
250 bp
NPT II
H
I
